# Memory-dependent effects on palatability in mice

**DOI:** 10.1016/j.physbeh.2016.09.001

**Published:** 2016-12-01

**Authors:** Joseph M. Austen, Jasmin A. Strickland, David J. Sanderson

**Affiliations:** Department of Psychology, Durham University, Science Site, South Road, Durham DH1 3LE, UK

**Keywords:** Learning, Memory, Negative contrast, Habituation, Neophobia, Palatability, Mice

## Abstract

While palatability depends on the properties of particular foods, it is also determined by prior experience, suggesting that memory affects the hedonic value of a substance. Here, we report two procedures that affect palatability in mice: negative contrast and flavour habituation. A microstructure analysis of licking behaviour was employed, with the lick cluster size (the number of licks made in quick succession before a pause) used as a measure of palatability. It was first confirmed that lick cluster size increased monotonically as a function of sucrose concentration, whereas consumption followed an inverted U-shaped function. In a successive negative contrast procedure it was found that when shifted from a high sucrose concentration (32%) to a low sucrose concentration (4%), mice made smaller lick clusters than a group that only received the low concentration. Mice exposed to flavours (cherry or grape Kool Aid) mixed with sucrose (16%) made larger lick clusters for familiar flavours compared to novel flavours. This habituation effect was evident after short (5 min) and long (24 h) test intervals. Both successive negative contrast and flavour habituation failed to affect levels of consumption. Collectively, the results show that prior experience can have effects on lick cluster size that are equivalent to increasing or decreasing the sweetness of a solution. Thus, palatability is not a fixed property of a substance but is dependent on expectation or familiarity that occurs as a result of memory.

## Highlights

• Consumption in mice is maximal with intermediate concentrations of sucrose.
• Lick cluster size increases monotonically as a function of sucrose concentration.
• A successive negative contrast procedure reduced lick cluster size.
• Flavour habituation led to an increase in lick cluster size.
• Memory has effects on palatability similar to altering the sweetness of a solution.

## 1 Introduction

Palatability reflects the hedonic value of foods and is a key determinant of feeding behaviour. Although it is determined by the properties of the food, it is also moderated by prior experience (e.g., [17]). While the level of intake of a particular food may reflect its palatability, it has been shown that measures of palatability are dissociable from measures of consumption. For example, dopaminergic manipulations affect levels of consumption, but not necessarily the orofacial taste reactivity responses [24] that are taken to reflect palatability responses [13], [20]. Similarly, there are manipulations that affect consumption, but have different effects on taste reactivity. For example, Pelchat, Grill, Rozin, and Jacobs [21] found that rats would avoid consuming flavours that had previously been paired with sickness and shocks to a similar extent, but only flavours that had been paired with sickness elicited negative taste reactions such as gaping and head shaking.

Given the distinct role of palatability in feeding behaviour it is important to understand both the psychological and neurobiological processes underlying palatability. Crucially, understanding of the neurobiological processes requires the use of animal models. Due to the prevalence of genetically modified mouse lines there is a benefit in identifying valid behavioural manipulations of palatability in mice. Currently, there are well-established behavioural procedures for examining palatability in rats, but there are fewer successful demonstrations in mice. Therefore, a purpose of the current study was to determine behavioural factors that affect palatability in mice by testing the effect of prior experience on consumption of sucrose solutions.

In order to assess palatability in mice we used a microstructure analysis of licking behaviour during consumption of sucrose. Rodents drink, typically, by making a series of licks in quick succession (a lick cluster) before a pause (e.g., [3], [5]). In rats the mean number of licks in a cluster increases monotonically as a function of sucrose concentration, whereas consumption follows an inverted U-shaped function [7], [23]. Therefore, lick cluster size has been proposed to provide a measure of palatability that is independent of levels of consumption (see [9], for a discussion). Consistent with this proposal, lick cluster size decreases with increasing concentration of unpalatable tastes (e.g., [14]). In the present study we used the mean lick cluster size as an alternative measure of palatability to the orofacial taste reactivity responses. While taste reactivity measures have been used to measure changes in palatability as a consequence of experience (e.g., [11]), the method requires human coding of the behaviours and surgery to enable the administration of substances directly into the oral cavity of rodents. Therefore, the measurement of lick cluster sizes avoids the use of those procedures.

We have previously demonstrated in mice that lick cluster size is affected by sucrose concentration, but this was with only a limited range of concentrations [1]. In addition it has been suggested that the monotonic effect of sucrose concentration on lick cluster size in mice is observed only when using a particularly large pause criterion (> 1 s) to determine the end of a lick cluster [15]. In order to validate the use of lick cluster size as a measure of palatability in mice Experiment 1 assessed consumption of a range of sucrose concentrations using a range of inter-lick cluster interval criteria.

The effect of memory on palatability was assessed using procedures that should either decrease or increase palatability. Experiment 2 examined a detrimental effect on palatability using a successive negative contrast procedure in which one group of mice was preexposed to 32% sucrose and another group was preexposed to 4% sucrose. Both groups were then allowed to consume 4% sucrose. In rats it has been demonstrated that the shift from a high concentration of sucrose to a low concentration results in a reduction in palatability of the low concentration of sucrose compared to a condition in which animals only experience the low concentration of sucrose [12]. In mice there are reports of negative contrast effects on levels of consumption (i.e., a shift from high to low concentration of sucrose results in reduced intake compared to controls, [19]), but there are few reports of an effect on palatability (see [1]).

A beneficial effect on palatability was examined using a flavour habituation procedure. A common finding in rats is that exposure to a novel flavour leads to a reduction in feeding that habituates with increased exposure [2]. In addition, measures of palatability increase as the flavour becomes familiar [16]. A flavour habituation effect on palatability was examined in Experiment 3 using a between-subjects procedure in which mice were exposed to a novel flavour and then after a short (5 min) delay half of the mice were exposed to the same flavour and the other half were exposed to a novel flavour. Experiment 4 examined the longer lasting effects of flavour habituation using a within-subjects procedure in which mice were exposed to one flavour over eight days and then given that flavour, and a novel flavour, 24 h after the last exposure.

## 2 Method

### 2.1 Subjects

Female C57BL/6 J/Ola mice obtained from Charles River, UK were used. Mice were caged in groups of four, in a temperature controlled housing room (light-dark cycle: 0800–2000). Mice in Experiment 1 were 10 weeks of age at the beginning of the experiment and weighed between 16.3 and 20.9 g (mean = 18.9 g). Mice in Experiment 2 were approximately five months old at the beginning of the experiment and weighed between 19.4 and 24.3 g (mean = 21.7 g). Mice in Experiment 3 were between 12 and 20 weeks of age at the beginning of the experiment and weighed between 14.1 and 25.7 g (mean = 21.4 g). Mice in Experiment 4 were between 16 and 27 weeks old and weighed between 17.4 and 24.5 g (mean = 20.0 g). Mice were initially allowed free access to food, but one week prior to training the weights of the mice were reduced, by receiving a restricted diet, and then subsequently maintained at 85% of their free-feeding weights. Mice were tested during the light period between 10 am and 4 pm. Throughout testing mice had ad libitum access to water in their home cages. All procedures were in accordance with the United Kingdom Animals Scientific Procedures Act (1986); under project license number PPL 70/7785.

### 2.2 Apparatus

A set of eight identical operant chambers (interior dimensions: 21.6 × 17.8 × 12.7 cm; ENV-307 W, Med Associates), enclosed in sound-attenuating cubicles (ENV-022 V, Med Associates) were used. The operant chambers were controlled by Med-PC IV software (Med Associates). The side walls were made from aluminium, and the front and back walls and the ceiling were made from clear Perspex. The chamber floors each comprised a grid of 24 stainless steel rods (0.32 cm diameter), spaced 0.79 cm apart and running perpendicular to the front of the chamber (ENV-307W-GFW, Med Associates). Retractable sippers (ENV-352AW, Med Associates) and a small hole in one wall of each chamber allowed graduated pipettes to be extended into, and retracted from, the chambers. The graduated pipette (0.1 ml) allowed measurement of consumption by comparing the volume before and after testing. Contact lickometer controllers (ENV-250, Med Associates) allowed contacts between the mice and the graduated pipettes to be recorded at a resolution of 0.01 s. A fan (ENV-025F, Med Associates) was located within each of the sound-attenuating cubicles and was turned on during sessions. Sucrose solutions were made weight/volume with commercially available sucrose in distilled water. For Experiments 3 and 4 the flavours used were cherry and grape Kool Aid (0.05% *w*/*v*, Kraft Foods USA, Rye Brook, NY, USA).

### 2.3 Procedure

#### 2.3.1 Experiment 1: the effect of sucrose concentration on licking behaviour

Mice ( N= 16) were allowed to consume 2.5%, 5%, 10% and 20% sucrose solution on four sessions, one session per day. Mice were presented with one of the concentrations per session, and the order in which the concentrations were presented was counterbalanced across mice. Specifically, half of the mice received the two low concentrations (2.5% and 5%) in the first two sessions and the remaining mice received the two high concentrations (10% and 20%). Within each of these groups the order of the concentrations in these first two sessions was counterbalanced. For the last two sessions mice received the two remaining concentrations in a counterbalanced order that across mice was also counterbalanced with respect to the order of the concentrations in the first two sessions. Sessions lasted 30 min and the pipette was extended into the chamber for the full duration of the session.

#### 2.3.2 Experiment 2: the effect of negative contrast on licking behaviour

Mice were randomly allocated to either group Unshift (N= 8) or group Shift (N = 8). The groups did not differ in their free-feeding weights (Unshift: 22.0 g; Shift: 21.0 g; *F*(1, 14) = 1.8, *p* = 0.21). Mice received eight training sessions, consisting of one trial per session, one session per day, in which a sucrose solution was available for consumption. Each trial lasted 15 min; however, the pipette was only extended into the chamber for the final ten minutes of the trial (similar to the procedure used by Austen and Sanderson [1]). Group Unshift received 4% sucrose solution on each training session, and were subsequently given a single test session 24 h after the final training session, using the same procedure as during training, in which they were also given 4% sucrose. Group Shift received 32% sucrose during training and then 4% sucrose in the test session.

#### 2.3.3 Experiment 3: the short-term effect of flavour habituation on licking behaviour

Mice were randomly allocated to either group Familiar (N= 16) or group Novel (N = 16). The groups did not differ in their free-feeding weights (Familiar: 21.5 g; Novel: 21.3 g; *F*(1, 30) < 1, *p* = 0.89). Mice received a single training trial in which they were allowed to consume 16% sucrose paired with a flavour. Five minutes later, mice received a single test trial, in which they could consume 16% sucrose paired with a flavour. For group Familiar the flavour during the test trial was the same as during the training trial. For group Novel the flavour during the test trial was different from the one during the training trial. For half of the mice within each group the flavour in the training trial was cherry and for the remaining mice it was grape. The mice in group Novel that received the cherry flavour during training received grape in the test trial, and vice versa for the remaining mice in group Novel. The training and test trials lasted fifteen minutes, with the pipette extended into the chamber for the entirety of this time.

#### 2.3.4 Experiment 4: the long-term effect of flavour habituation on licking behaviour

Mice (N= 16) initially received eight sessions of training, one session per day, in which they were allowed to consume 16% sucrose paired with a flavour. For half of the mice the flavour that was presented throughout training was cherry, with the remaining mice receiving grape. Each session consisted of two trials of fifteen minutes, with a ten minute ITI. In contrast to Experiment 3 the pipette was only extended into the chamber for the final ten minutes of each trial. Given that long-term habituation has been proposed to be context-dependent [26] this procedure was used to allow mice exposure to the context cues prior to the start of consumption in each session (see [4]). Twenty-four hours after the last session of training, mice received a single test session. On this test session, which consisted of two trials in the same manner as during training, mice were allowed to consume 16% sucrose paired with cherry during one trial and grape during the other. Half of the mice received the same flavour as during training for the first trial of the test session, with the remainder receiving the novel flavour first.

### 2.4 Data and statistical analyses

For all experiments three aspects of licking behaviour were measured: total number of licks, mean number of licks per cluster (lick cluster size) and amount of sucrose solution consumed (ml). A lick cluster was defined as a series of two or more licks made with < 0.5 s between the end of one lick and the start of the next. For Experiment 1, in order to assess the sensitivity of the measure with a range of criteria, additional analyses were conducted using lick cluster criteria of < 0.25 s and < 1 s between licks. For the crucial test phases of Experiments 2–4 licking was analysed in time bins. The test phase of Experiment 2 was analysed in five 2-min time bins in order to make comparisons with another study of negative contrast reported by Austen and Sanderson [1] in which there was an effect of negative contrast on lick cluster size in the initial 2-min time bin. The test phase of Experiment 3, in which flavour habituation was examined, was analysed in three 5-min time bins. Experiment 4 also examined flavour habituation, but the test phase, in contrast to Experiment 3, lasted only 10 min (see procedural details). Therefore, in order to analyse flavour habituation in a similar manner across experiments, Experiment 4 was analysed in two 5-min time bins. For each time bin lick cluster size was calculated by dividing the total number of licks made within clusters of licks in that time bin by the number of lick clusters completed within the time bin. This method approximates the mean lick cluster size for a particular time bin, but potentially leads to a mean that differs to an extent from the mean of the lick clusters that were started and completed within the time bin. All data were analysed using one-way or multifactorial ANOVA. Interactions were analysed with simple main effects analysis using the pooled error term from the original ANOVA, or separate repeated measures ANOVA for within-subject factors with more than two levels. Where sphericity of within-subjects variables could not be assumed, a Greenhouse-Geisser correction was applied to produce more conservative *p*-values.

## 3 Results

### 3.1 Experiment 1: the effect of sucrose concentration on licking behaviour

#### 3.1.1 Total licks

**Fig. 1:**
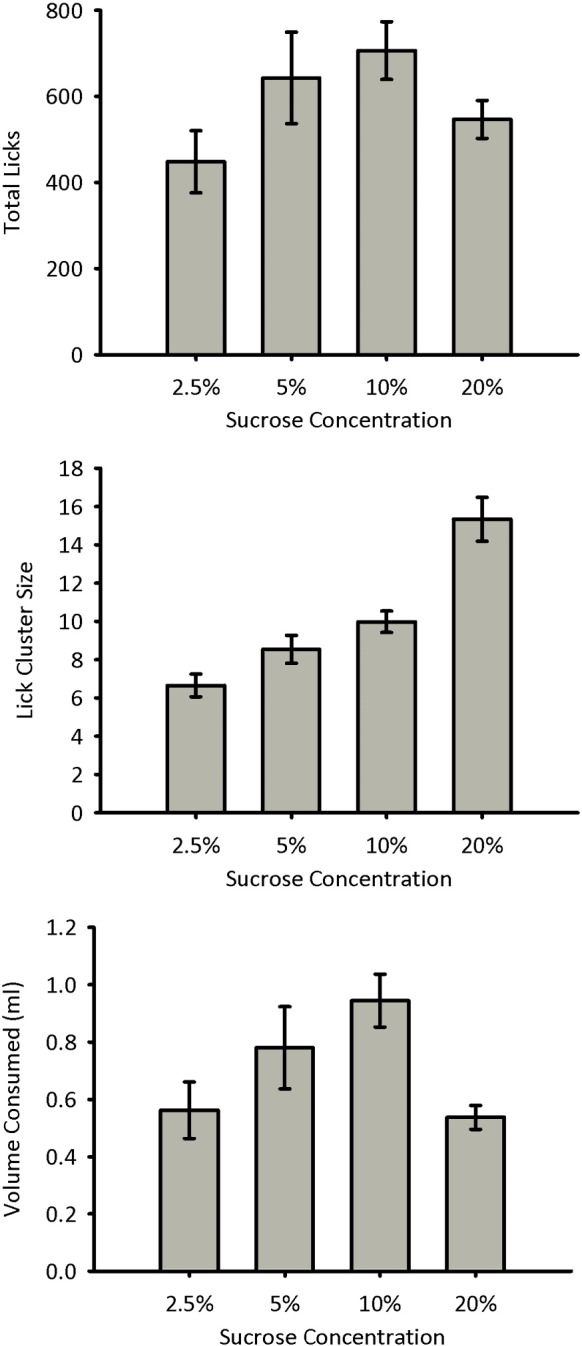
Data for Experiment 1. Total number of licks (top panel), mean lick cluster size (centre panel), and volume of sucrose consumed (bottom panel) are shown for each of the four sucrose concentrations. Error bars indicate ± SEM.

The total number of licks for the four sucrose concentrations is shown in Fig. 1 (top panel). The number of licks increased with concentration from 2.5% to 10% sucrose, but was lower for 20% sucrose than for both 10% and 5%. A repeated-measures ANOVA of concentration failed to show a significant effect of sucrose concentration on total licks, *F*(3, 45) = 2.73, *p* = 0.10.^1^ Trend analysis, however, showed a significant quadratic trend between concentration and total licks, *F*(1, 15) = 24.3, *p* < 0.001, but no significant linear trend, *F*(1, 15) = 0.83, *p* = 0.38.

#### 3.1.2 Lick cluster size

The mean lick cluster size during consumption of the four sucrose concentrations, using a lick cluster criterion of < 0.5 s between licks, is shown in Fig. 1 (centre panel). The lick cluster sizes showed a monotonic increase with increasing sucrose concentration. A repeated measures ANOVA of concentration showed a significant effect of concentration, *F*(3, 45) = 37.8, *p* < 0.001. In addition, trend analysis showed a significant linear trend between sucrose concentration and lick cluster size, *F*(1, 15) = 72.5, *p* < 0.001. Additional analyses were carried out using criteria of < 0.25 s and < 1 s between licks (data not shown). It was found that 85% of lick clusters that were separated by at least 0.25 s were also separated by at least 0.5 s, and 91% of lick clusters that were separated by at least 0.5 s were also separated by at least 1 s. Similar to the results found using the < 0.5 s criterion, a monotonic increase in lick cluster size with increasing sucrose concentration was found with the < 0.25 s and < 1 s criteria (< 0.25 s: F(3,45) = 19.9, *p* < 0.001; < 1 s: F(3,45) = 42.0, p < 0.001). Comparison of the effect sizes revealed that there was little difference between the 0.5 and 1 s criteria (partial eta squared equalled 0.72 and 0.74 respectively), but the 0.25 s criterion produced the lowest effect size (partial eta squared equalled 0.57). Analyses in the subsequent experiments used the 0.5 s criterion similar to that commonly used in rat studies (e.g., [7], [8]).

#### 3.1.3 Consumption

The volume of each sucrose solution consumed is shown in Fig. 1 (bottom panel). Similar to the pattern of results for total licks, the volume consumed increased with concentration from 2.5% to 10% sucrose, but was lower for 20% sucrose than all other concentrations. Consumption data was lost for one animal in the 5% sucrose condition; therefore the statistical analyses represent data from 15 animals. A repeated measures ANOVA of concentration showed a significant effect of concentration, *F*(3, 42) = 4.87, *p* = 0.029. Post-hoc analysis of the effect of concentration using the Bonferroni correction for multiple comparisons revealed that mice consumed less of 2.5% sucrose than 5% and 10% (*p*-values < 0.05), but not 20% sucrose (*p* > 0.9). Mice consumed less of 20% sucrose than 10% sucrose (*p* < 0.001). In addition, trend analysis showed a significant quadratic trend between consumption and concentration, *F*(1, 15) = 57.1, *p* < 0.001, but no significant linear trend, *F*(1, 15) = 0.18, *p* = 0.68.

### 3.2 Experiment 2: the effect of negative contrast on licking behaviour

#### 3.2.1 Training

Across training sessions group Shift, exposed to 32% sucrose, made a significantly greater number of licks per session than group Unshift, which was exposed to 4% sucrose (Shift mean = 748 ± 53 SEM; Unshift mean = 561 ± 53 SEM; *F*(1, 14) = 6.29, *p* = 0.025). Group Shift also made significantly larger lick clusters (Shift mean = 20.5 ± 1.9 SEM; Unshift mean = 14.3 ± 1.5 SEM; *F*(1, 14) = 6.58, *p* = 0.022). Although group Shift consumed more than group Unshift across sessions, this difference failed to reach significance (Shift mean = 0.80 ml ± 0.04 SEM; Unshift mean = 0.68 ml ± 0.04 SEM; *F*(1, 14) = 3.73, *p* = 0.074).

#### 3.2.2 Test: total licks

**Fig. 2:**
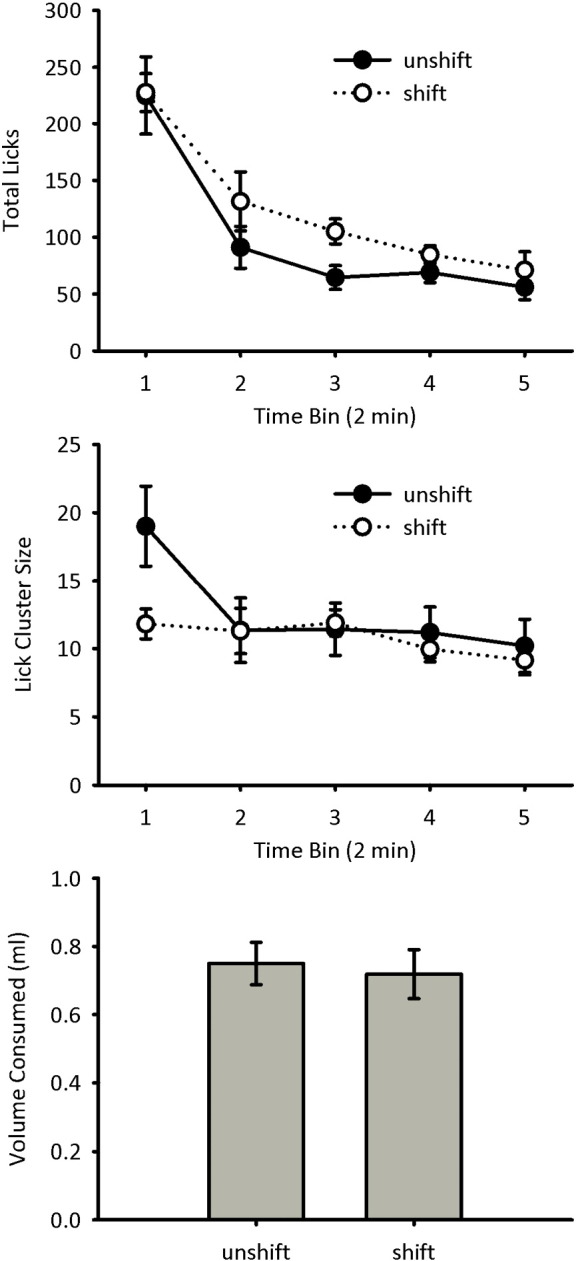
Test data for Experiment 2. Total number of licks (top panel) and mean lick cluster size (centre panel) are shown in two-minute time bins for each group. The amount of sucrose solution consumed by each of the two groups during the test trial is shown in the bottom panel. Error bars indicate ± SEM.

Licking during the test session was analysed in five two-minute time bins. The total number of licks of 4% sucrose made during the test session by mice in groups Shift and Unshift are shown in Fig. 2 (top panel). Licking decreased over time for both groups, with the number of licks being numerically higher for group Shift than Unshift. A mixed-model ANOVA of bin x group showed a significant main effect of bin, *F*(4, 56) = 46.3, *p* < 0.001, but no significant main effect of group, *F*(1, 14) = 1.54, *p* = 0.24, and no significant interaction between bin and group, *F*(4, 56) = 0.75, *p* = 0.50.

#### 3.2.3 Test: lick cluster size

The lick cluster sizes for groups Shift and Unshift are shown in Fig. 2 (centre panel). Group Unshift initially showed a greater mean lick cluster size than group Shift, but by the second time bin lick cluster sizes for the two groups were similar. This was due to a reduction in lick cluster size over time for group Unshift. A mixed-model ANOVA of bin x group showed a significant main effect of bin, *F*(4, 56) = 6.38, *p* < 0.001, but no significant main effect of group, *F*(1, 14) = 0.82, *p* = 0.38. However, there was a significant interaction between bin and group, *F*(4, 56) = 3.16, *p* = 0.021. Simple main effects analysis of the interaction showed that lick cluster size was higher for group Unshift than for group Shift during the first two minute bin, *F*(1, 14) = 5.27, *p* = 0.038, but not during any other bins, *F-*values < 0.4, *p-*values > 0.5. The lick cluster size for group Unshift decreased over the course of the test session, *F*(4, 28) = 6.29, *p* = 0.001, but this was not the case for group Shift, *F*(4, 28) = 1.52, *p* = 0.22.

#### 3.2.4 Test: consumption

The volume of 4% sucrose solution consumed during the test session by mice in groups Shift and Unshift is shown in Fig. 2 (bottom panel). The two groups consumed a similar amount, *F*(1, 14) = 0.11, *p* = 0.75.

### 3.3 Experiment 3: the short-term effect of flavour habituation on licking behaviour

#### 3.3.1 Training

The mean number of licks during the training sessions was 559 (± 38 SEM), the mean lick cluster size was 22.1 (± 1.4 SEM), and the mean consumption was 0.48 ml (± 0.03 SEM). There were no significant differences between group Familiar and group Novel during the training stage on any of the three measures, *F*-values ≤ 1, *p*-values > 0.3.

#### 3.3.2 Test: total licks

**Fig. 3:**
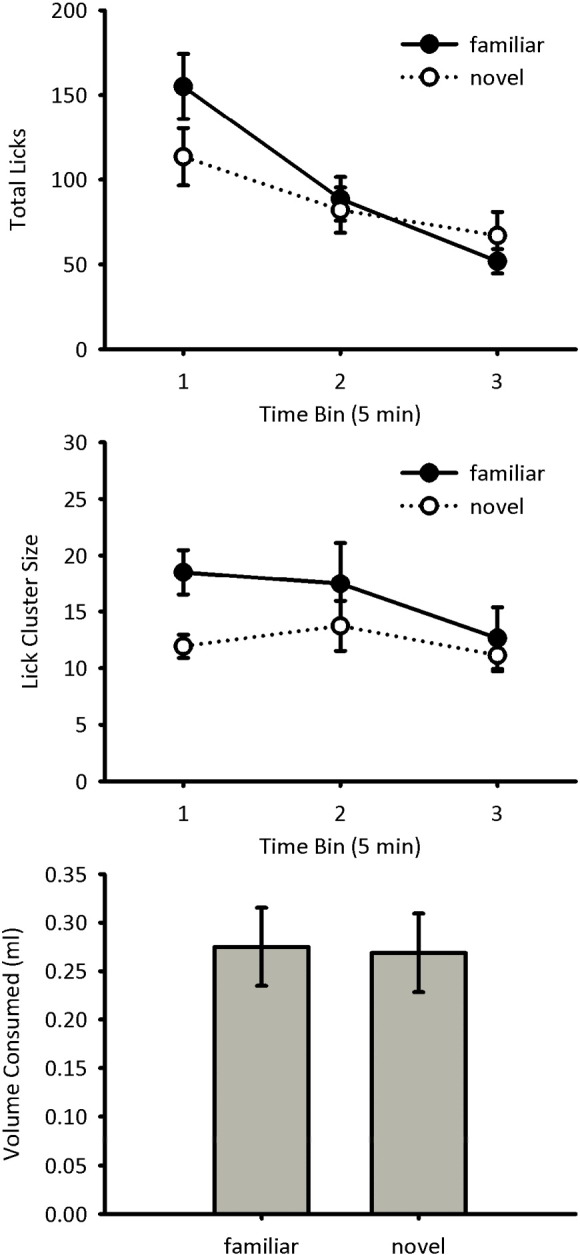
Test data for Experiment 3. Total number of licks (top panel) and mean lick cluster size (centre panel) are shown in five-minute time bins for each group. The amount of sucrose solution consumed by each of the two groups during the test trial is shown in the bottom panel. Error bars indicate ± SEM.

Licking during the test trial was analysed in three five-minute bins. The total numbers of licks made by group Familiar and group Novel are shown in Fig. 3 (top panel). The number of licks decreased over the course of the trial for both groups, with more licks made by group Familiar than group Novel at the beginning of the trial and fewer licks made by group Familiar than group Novel at the end. A mixed-model ANOVA of bin x group showed a significant main effect of bin, *F*(2, 60) = 22.4, *p* < 0.001, but no significant main effect of group, *F*(1, 30) = 0.50, *p* = 0.48. The interaction between these main effects failed to reach significance, *F*(2, 60) = 3.17, *p* = 0.060. Given that the novel flavour will become increasingly familiar across the test trial the difference between the groups would be anticipated to be greatest earlier in the test trial. Therefore, a second analysis was conducted restricted to just the first time bin. It was found, however, that the effect of novelty was not significant, *F*(1, 30) = 2.64, *p* = 0.12.

#### 3.3.3 Test: lick cluster size

The lick cluster sizes for group Familiar and group Novel during the test trial are shown in Fig. 3 (centre panel). The lick cluster sizes were higher for group Familiar than for group Novel across all three time bins, although this difference was more marked at the beginning of the trial. A mixed-model ANOVA of bin x group showed no significant main effect of bin, *F*(2, 60) = 1.98, *p* = 0.15, and no interaction between bin and group, *F*(2, 60) = 0.76, *p* = 0.47. The main effect of group was not significant, *F*(1, 30) = 2.97, *p* = 0.095. Given that the novel flavour will become increasingly familiar across the test trial the difference between the groups would be anticipated to be greatest earlier in the test trial. Therefore, when the analysis was restricted to the first time bin it was found that Group Novel made significantly lower lick cluster sizes than group Familiar, *F*(1, 30) = 8.66, *p* = 0.006.

#### 3.3.4 Test: consumption

The amount of sucrose consumed by groups Novel and Familiar during the test trial is shown in Fig. 3 (bottom panel). Consumption levels were similar between the two groups. A between-subjects ANOVA of group showed no significant main effect, *F*(1, 30) = 0.01, *p* = 0.91.

### 3.4 Experiment 4: the long-term effect of flavour habituation on licking behaviour

#### 3.4.1 Training

The mean number of licks during the training sessions was 864 (± 84 SEM), the mean lick cluster size was 27.9 (± 3.5 SEM), and the mean consumption was 0.78 ml (± 0.03 SEM). The number of licks and mean lick cluster size remained similar across sessions, *F*-values < 1.4, *p*-values > 0.27, but the amount consumed showed a general increase over sessions, *F*(7, 105) = 14.5, *p* < 0.001.

#### 3.4.2 Test: total licks

**Fig. 4:**
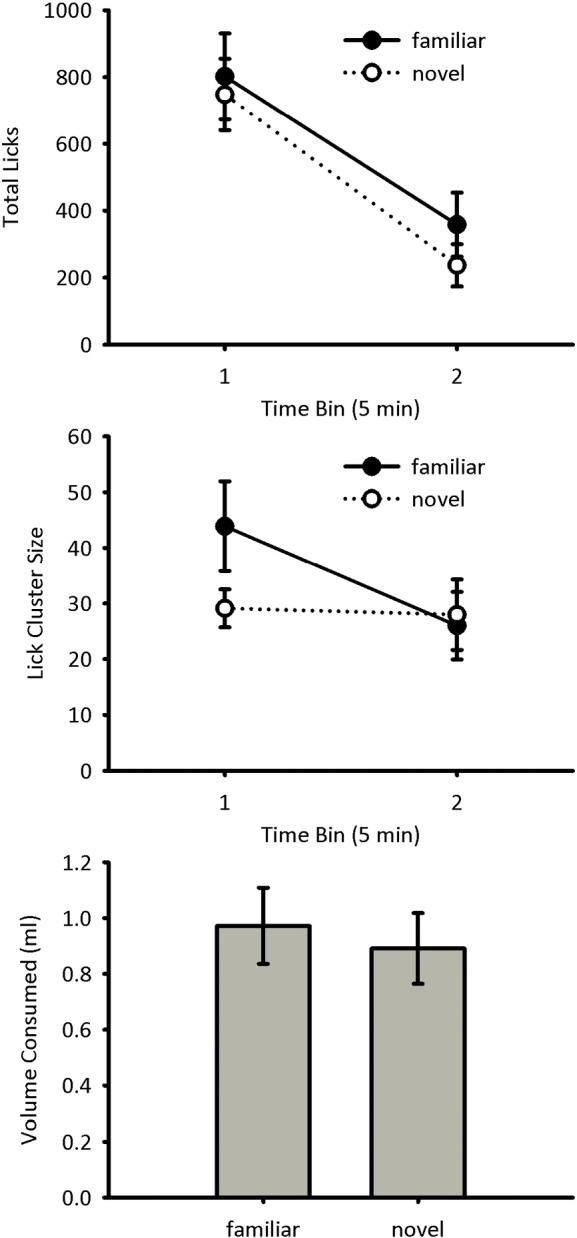
Test data for Experiment 4. Total number of licks (top panel) and mean lick cluster size (centre panel) are shown in five-minute time bins for the familiar and novel flavours. The amount of sucrose solution consumed during each test trial is shown in the bottom panel. Error bars indicate ± SEM.

Licking during the test session was analysed in two five-minute bins. The total number of licks made during the test session to the novel and familiar flavours is shown in Fig. 4 (top panel). The number of licks decreased across the course of the session for both novel and familiar flavours, and there were numerically more licks to the familiar flavour than to the novel flavour. A repeated measures ANOVA of bin x novelty showed a significant main effect of bin, *F*(1, 15) = 125, *p* < 0.001, but no significant main effect of novelty, *F*(1, 15) = 0.33, *p* = 0.58, and no interaction between bin and novelty, *F*(1, 15) = 0.80, *p* = 0.39.

#### 3.4.3 Test: lick cluster size

The lick cluster sizes made during the test session to the novel and familiar flavours are shown in Fig. 4 (centre panel). Lick cluster size could not be calculated for one mouse during the second bin of the test session due to the mouse failing to make any licks during this period. To prevent this mouse from being excluded from the statistical analysis, its lick cluster size was assumed to be the group mean for this time bin. Lick cluster sizes decreased across the session for the familiar flavour, but not the novel flavour, with higher lick cluster sizes to the familiar flavour than to the novel flavour early in the test session. A repeated measures ANOVA of bin x novelty showed a significant main effect of bin, *F*(1, 15) = 11.8, *p* = 0.004, but no significant main effect of novelty, *F*(1, 15) = 3.26, *p* = 0.091. There was a significant interaction between the two main effects, *F*(1,15) = 5.08, *p* = 0.040. Simple main effects analysis of this interaction showed that the lick cluster size for the familiar flavour was higher than the novel flavour in the first time bin, *F*(1, 15) = 6.26, *p* = 0.024, but not in the second time bin, *F*(1, 15) = 0.22, *p* = 0.65. Lick cluster size decreased for the familiar flavour over the course of the test session, *F*(1, 15) = 12.1, *p* = 0.003, but this was not the case for the novel flavour, *F*(1, 15) = 0.08, *p* = 0.79.

#### 3.4.4 Test: consumption

The volumes of sucrose consumed during the test session to the novel and familiar flavours are shown in Fig. 4 (bottom panel). The amount of sucrose consumed was similar between the two conditions. A repeated measures ANOVA of novelty showed no significant main effect, *F*(1, 15) = 0.11, *p* = 0.75.

## 4 Discussion

Increasing the sucrose concentration of a solution produced a monotonic increase in the size of the lick clusters made during consumption of that solution. It was found that the negative contrast procedure and habituation of neophobia procedures affected lick cluster size in a manner that was similar to that caused by decreasing or increasing, respectively, the concentration of sucrose. These results demonstrate that memory for prior consumption of food can have either a positive or negative effect on palatability depending on the particular procedure used.

Experiment 1 confirmed that lick cluster size provides an effective measure of palatability in mice that is dissociable from levels of consumption. It was found that the number of licks within a cluster increased monotonically as a function of the concentration of sucrose. In contrast, the total number of licks and the volume of sucrose solution consumed followed an inverted U-shaped function, with moderate sucrose concentrations producing a greater number of licks and volume consumed than either low or high concentrations. In contrast to a study by Johnson et al. [15] we found that C57BL6 mice showed an effect of sucrose concentration on lick cluster size using a variety of pause criteria (i.e., 0.25, 0.5 and 1 s), suggesting that there are not considerable differences between these criteria with the majority of pauses between clusters of licks that lasted 0.25 s also lasting at least 1 s. In the study by Johnson et al. [15] there was an effect of sucrose concentration on lick cluster size only when a 1 s pause criterion was used, suggesting that pauses shorter than 1 s may reflect interruptions in licking that are not related to the palatability of the solution consumed. It is likely that the discrepancy between the current results and those from the Johnson et al. [15] study is due to differences in the methods used to measure licking behaviour. In the current study mice were allowed to consume sucrose solutions from a pipette, such that the flow of the solution was dependent on the tongue making contact with the pipette. In the study by Johnson et al. [15] mice were able to lap sucrose solutions that were periodically pumped into a food well. Therefore, the contrasting results may reflect differences in the lick clusters made when lapping sucrose versus licking from a pipette. Importantly, our results parallel those of a study in rats [23] that demonstrated that lick cluster sizes, as determined by a relatively short pause criterion (0.3 s) increase monotonically as a function of sucrose concentration. In this study rats drank by licking from a spout. Collectively these results may suggest that the lapping procedure used by Johnson et al. [15] lacks the sensitivity to detect changes in cluster size at short pause criteria.

A successive negative contrast effect on lick cluster size was found when mice were shifted from a high sucrose concentration to a low concentration. The reduction in lick cluster size, compared to the unshifted control group, was transient, lasting for only the first two minutes of the ten minute test trial. In contrast, there was no significant effect on the number of licks or the volume of sucrose solution consumed. The lack of effect on consumption is surprising given that it is commonly found in studies with rats (see [10], for a discussion). A successive negative contrast effect has been reported in mice [19], however, in contrast to the present study, mice received greater exposure to the high concentration prior to the shift to the lower concentration. Therefore, it is possible that our procedure is suboptimal for producing a negative contrast effect on consumption. Nonetheless, we have found a similar pattern of results using a within-subjects design [1]. In that study, mice were exposed to 32% sucrose in one context and 4% in another context. In the critical test mice received 4% sucrose in both contexts. Similar to the current results, mice showed a transient reduction in lick cluster size in the context in which they had previously experienced 32% sucrose, but there was no overall effect on consumption. Therefore, it is likely that the negative contrast effect on lick cluster size in Experiment 2 was caused by context-dependent memory. The fact that memory retrieval of the high sucrose concentration had a negative rather than a positive effect on palatability may reflect habituation, potentially as a result of conditioned diminution of the unconditioned response [25]. Importantly, the between-subjects demonstration of a negative contrast effect on palatability in the current study makes it unlikely that the negative contrast effect in the Austen and Sanderson [1] study was an artefact of the within-subjects procedure that was used. For example, the between-subjects effect makes it unlikely that the within-subject effect depended on differential conditioning of the contexts that could result in the context paired with 4% sucrose becoming a conditioned inhibitor of 32% sucrose.

A flavour habituation effect on lick cluster size was found when mice were preexposed to a flavour. Similar to the results for the negative contrast study, there was no overall effect on levels of consumption. This was true when mice received a brief preexposure and were tested after a short interval (Experiment 3) and when mice received extensive preexposure and were tested after a long interval (Experiment 4). The procedures used in the two demonstrations of flavour habituation rule out two potential accounts of the effect on lick cluster size. First, the larger lick cluster size for the familiar flavour does not simply reflect a general enhancement in licking behaviour. Both experiments used a stimulus specific test of habituation, comparing the response to the familiar flavour with that for a novel flavour, ruling out nonspecific changes in behaviour. Therefore, the increase in lick cluster size was specific to the preexposed, familiar flavour. Second, the flavour habituation effect was evident after a relatively long, 24-h, interval making it unlikely that short-term sensory adaptation can account for the results. While it is possible that performance in the short interval test reflects habituation caused by the short-term memory, it is also possible that it reflects to some extent sensory adaptation. Sensory adaptation would, however, likely recover over a 24-h period. Due to the stimulus-specific and long-term nature of the effect, the increase in lick cluster size likely reflects a weakening of the unconditioned, phobic response to the flavour due to memory retrieval.

In contrast to other tests of habituation of neophobia in feeding behaviour (e.g., [16]), we failed to find an effect on consumption. This suggests that the increase in consumption during preexposure that was observed in Experiment 4 was not due to stimulus-specific habituation. Other studies have, however, found stimulus-specific effects on consumption (e.g., [22]). The lack of effect in the current study may be due to the flavours that were used being relatively palatable independent of the amount of preexposure, which may have resulted in low levels of neophobia. Regardless of the reasons for failing to find an effect on consumption the results may suggest that palatability is a more sensitive measure of habituation and although animals may readily consume novel flavours, they are perceived as less palatable than familiar flavours.

The effects of negative contrast and flavour habituation were transient, being evident at the beginning of the test phase, but not by the end. Two factors are likely to have contributed to the transient nature of the effects. First, licking typically decreased over the test sessions such that differences between conditions may have been harder to detect in the latter portions of the test due to a floor effect. Second, the effects of negative contrast and flavour habituation are likely to reduce over the course of the test phase. Thus, in the case of negative contrast, the effect of the down-shift in sucrose concentration is likely to be greatest initially. Similarly, in the case of flavour habituation, the effect of novelty will reduce as the novel flavour becomes increasingly familiar over the test phase. In Experiment 2 it was found that there was an effect of negative contrast on lick cluster size in the initial 2-min time bin, but not thereafter. As mentioned previously, this effect is similar to that found in a previous study of negative contrast [1], suggesting that the effect of negative contrast on palatability does not last past the first two minutes of consumption. Due to differences in the procedural details, Experiments 3 and 4 were analysed in five-minute time bins. For these experiments, however, it was also found that the effect of flavour habituation was evident in the first time bin, but not thereafter. It remains to be determined the degree to which the lack of differences between conditions in the latter portions of the test phase is due to a reduction in licking caused by satiety or by extinction of the effects of the experimental manipulations.

## 5 Conclusions

The results of the present set of experiments demonstrate behavioural procedures for manipulating the palatability of sucrose in mice, suggesting that memory plays an important role in the hedonic value of foods. These procedures will be useful for examining the neural basis of cognitive factors in feeding behaviour. The results also provide further evidence that consumption and palatability are dissociable. Therefore, while initial consumption is linked to the palatability of a substance (e.g., [6]), overall levels of consumption provide little information about palatability. Thus, lick cluster size provides a measure of palatability that is more informative for models of anhedonia (e.g., [18]) than consumption alone.

## Footnotes

1 An additional analysis was conducted in which the counterbalancing factor of whether mice received the two higher concentrations first or last was included. It was now found that the effect of concentration was significant (*F*(3, 42) = 9.2, *p* < 0.001). Adding this counterbalancing factor to the analysis of mean lick cluster size and consumption still resulted in revealing significant effects of concentration.
